# THE DAWN OF A SOCIETY OF DIVORCEES: CHANGING PATTERNS OF LATE LIFE CIVIL STATUS

**DOI:** 10.1093/geroni/igac059.155

**Published:** 2022-12-20

**Authors:** Peter Öberg, Torbjörn Bildtgård

**Affiliations:** University of Gävle, Gävle, Gavleborgs Lan, Sweden; Stockholms University, Stockholm, Stockholms Lan, Sweden

## Abstract

Half a century ago Lopata used the concept “society of widows” to describe the gendered reality of late life singlehood, where widowed women were excluded from coupled social life, depended on a community of other widows for social integration, and refrained from initiating new relationships due to “sanctification” of their former husbands. We use Swedish, American and EU census data 1970-2020 and a national survey to Swedes 60-90 years old (n=1225; response rate 42%) to illustrate a substantial change in the demographic landscape of late life civil status. More people enter later life as divorcees or become divorced at a high age. Among Swedes 60+ divorcees outnumber widowed people, and the incidence of late life divorce has more than doubled since the millennium in what has been called the “grey divorce revolution”. Many other Western countries follow the same demographic trend, posing important questions about the transformation of unmarried later life. We conclude by proposing the concept “society of divorcees” for this new demographic landscape of late life singlehood, argue that research is needed to capture this new reality, and discuss the implications of this change for access to social support later life.

